# BED: a Biological Entity Dictionary based on a graph data model

**DOI:** 10.12688/f1000research.13925.3

**Published:** 2018-07-19

**Authors:** Patrice Godard, Jonathan van Eyll

**Affiliations:** 1 Clarivate Analytics, Carlsbad, CA, 92008, USA; 2 UCB Pharma, Braine-l’Alleud, 1420, Belgium

**Keywords:** genomics, transcriptomics, proteomics, RNA-seq, microarray, database, identifiers

## Abstract

The understanding of molecular processes involved in a specific biological system can be significantly improved by combining and comparing different data sets and knowledge resources. However, these information sources often use different identification systems and an identifier conversion step is required before any integration effort. Mapping between identifiers is often provided by the reference information resources and several tools have been implemented to simplify their use. However, most of these tools do not combine the information provided by individual resources to increase the completeness of the mapping process. Also, deprecated identifiers from former versions of databases are not taken into account. Finally, finding automatically the most relevant path to map identifiers from one scope to the other is often not trivial. The Biological Entity Dictionary (BED) addresses these three challenges by relying on a graph data model describing possible relationships between entities and their identifiers. This model has been implemented using Neo4j and an R package provides functions to query the graph but also to create and feed a custom instance of the database. This design combined with a local installation of the graph database and a cache system make BED very efficient to convert large lists of identifiers.

## Introduction

Since the advent of genome sequencing projects, many technologies have been developed to get access to different molecular information on a large scale and with high throughput. DNA micro-arrays are probably the archetype of such technology because of their historical impact on gathering data related to nucleic acids: genomic DNA and RNA. They triggered the emergence of “omics” fields of research such as genomics, epigenomics or transcriptomics. Lately massive parallel sequencing further increased the throughput of data generation related to nucleic acids by several orders of magnitude. In a different way, mass spectrometry-related technologies allow the identification and the quantification of many kinds of molecular entities such as metabolites and proteins. Many information systems have been developed to manage the exploding amount of data and knowledge related to biological molecular entities. These resources manage different aspects of the data. For example, some are genome or proteome centered, whereas others are focused on molecular interactions and pathways. Thus, all these resources rely on different identifier systems to organize concepts of their interest. The value of all the experimental data and all the knowledge collected in public or private resources is very high as such but is also often synergistically leveraged by their cross comparison in a dedicated manner. Indeed, many data sets can be relevant when addressing the understanding of a specific biological system, a phenotypic trait or a disease for example. These data sets can focus on different biological entities such as transcripts or proteins in different tissues, conditions or organisms. Comparing all these data and integrating them with available knowledge requires the ability to map the identifiers on which each resource relies.

To achieve this task public and proprietary information systems provide mapping tables between their own identifiers (ID) and those from other resources. Furthermore, many tools have been developed to facilitate the access to this information. Ensembl BioMart ( Kinsella *et al.*, 2011), mygene ( Wu *et al.*, 2013), and g:Profiler ( Reimand *et al.*, 2016a) are popular examples among many others. These resources are convenient and easy to use with information managed by their maintainers. However, in general (BioMart provides tools to configure marts), this information cannot be easily customized, optimized or extended by an empowered user according to his knowledge or to internal, non-public or non-standard data. Recognizing these challenges van Iersel *et al.* (2010) proposed the BridgeDb framework providing to bioinformatics developers a standard interface between tools and mapping services and also allowing the easy integration of custom data by a transitivity mechanism. However, to our knowledge, transitivity in the BridgeDb framework is not leveraged to improve the completeness of ID conversion (e.g. the number of Ensembl gene ID actually converted to Entrez gene ID).

Here we present BED: a biological entity dictionary. BED has been developed to address three main challenges. The first one is related to the completeness of identifier mappings. Indeed, direct mapping information provided by the different systems are not always complete and can be enriched by mappings provided by other resources. More interestingly, direct mappings not identified by any of these resources can be indirectly inferred by using mappings to a third reference. For example, many human Ensembl gene ID are not directly mapped to any Entrez gene ID but such mappings can be inferred using respective mappings to HGNC ID. The second challenge is related to the mapping of deprecated identifiers. Indeed, entity identifiers can change from one resource release to another. The identifier history is provided by some resources, such as Ensembl or the NCBI, but it is generally not used by mapping tools. The third challenge is related to the automation of the mapping process according to the relationships between the biological entities of interest. Indeed, mapping between gene and protein ID scopes should not be done the same way than between two scopes regarding gene ID. Also, converting identifiers from different organisms should be possible using gene orthologs information.

To meet these challenges, we designed a graph data model describing possible relationships between different biological entities and their identifiers. This data model has been implemented with the Neo4j ^®^ graph database ( Neo4j inc, 2017). Graph databases are very efficient to implement biological data models. They are more and more used in different fields of application. For example, they are successfully used for integrating various pieces of knowledge ( Pareja-Tobes *et al.*, 2015; Yoon *et al.* (2017)), describing disease and phenotype relationships ( Pareja-Tobes *et al.*, 2015) or modeling molecular networks and pathways ( Dai *et al.*, 2016; Fabregat *et al.* (2018)).

In addition to the Neo4j ^®^ graph database, conversion rules have been defined and coded in an R ( R Core Team, 2017) package. A particular attention has been put on the efficiency of the tool by implementing a cache system making recurrent queries fast.

Finally, we provide, for convenience, an instance of the BED database focused on human, mouse and rat organisms. Nevertheless, many functions are available in the R package to customize this instance or to construct other instances tailored to other needs.

## Methods

### Data model

**Figure 1.**
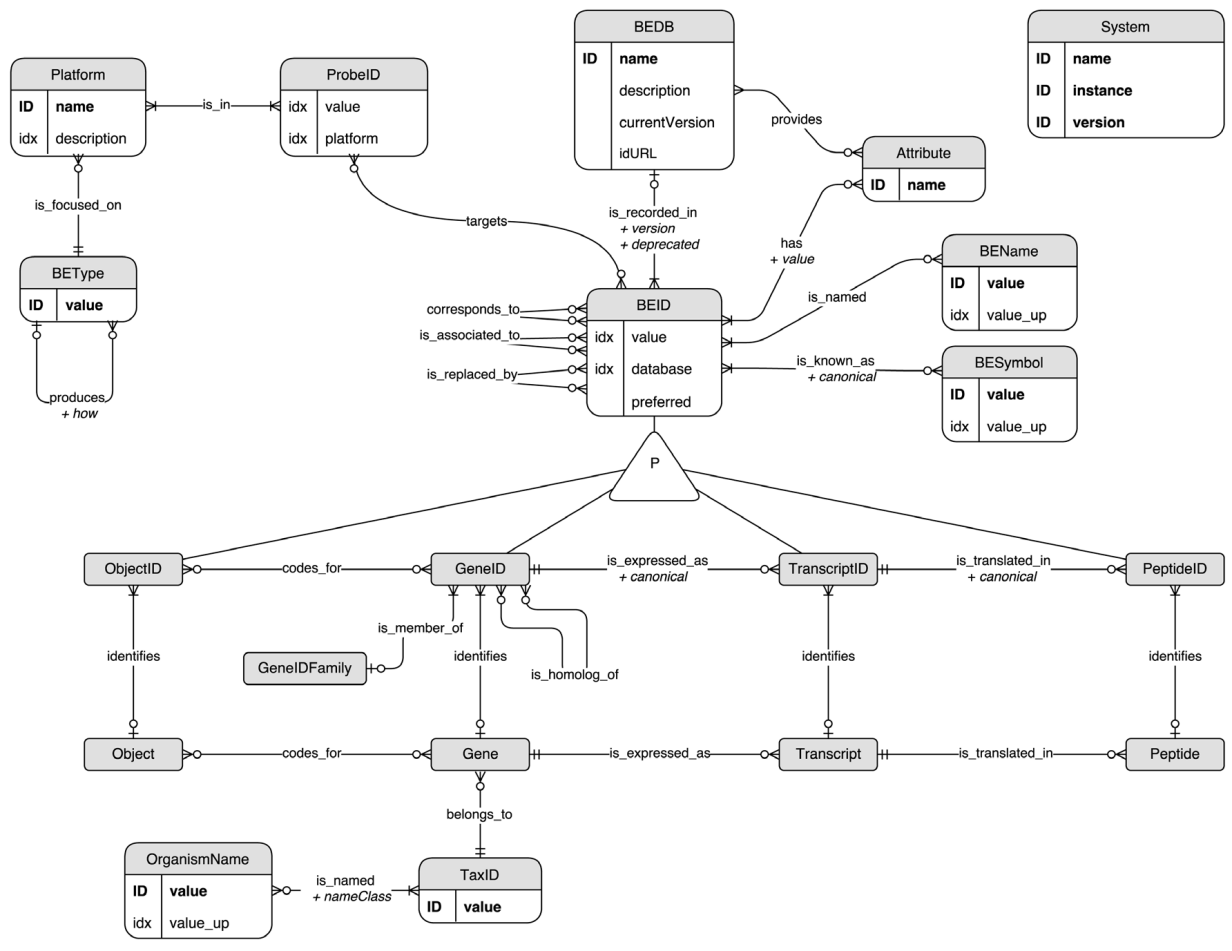
The BED graph data model. The model is shown as an Entity/Relationship (ER) diagram: entities correspond to graph nodes and relationships to graph edges. “ID” and “idx” indicate if the corresponding entity property is unique or indexed respectively. Some redundancies occur in this data model. Indeed some “value” properties are duplicated in upper case (“value_up”) in order to improve the performance of case-insensitive searches. Also, the database of a BEID node is provided as a property to ensure uniqueness of the couples of “database” and “value” properties. The same approach has been applied for the “platform” property of ProbeID nodes.

The BED (Biological Entity Dictionary) system relies on a data model inspired by the central dogma of molecular biology ( Crick, 1970) and describing relationships between molecular concepts usually manipulated in the frame of genomics studies ( Figure 1). A biological entity identifier ( *BEID*) can identify either a *Gene* ( *GeneID*), a *Transcript* ( *TranscriptID*), a *Peptide* ( *PeptideID*) or an *Object* ( *ObjectID*). *Object* entities can correspond to complex concepts coded by any number of genes (i.e. a protein complex or a molecular function). *BEID* are extracted from public or private databases ( *BEDB*). *BEDB* can provide an *Attribute* related to each *BEID*. For example, it can be the sequencing region provided by the Ensembl database ( Zerbino *et al.*, 2018) or the identifier status provided by Uniprot ( The UniProt Consortium, 2017). *BEID* can have one or several associated names ( *BENames*) and symbols ( *BESymbol*). *GeneID* can have one or several homologs in other organisms belonging to the same *GeneIDFamily*. Many genomics platforms, such as micro-arrays, allow the identification of biological entities by using probes identified by *ProbeID*. In general, *BEID* can be targeted by several probes belonging to a *Platform* which is focused on one, and only one, type of entity (BEType) among those described above: *Gene*, *Transcript*, *Peptide* or *Object*.

According to this data model, the scope of an identifier is defined by three features:

- its type: a *BEType* or “Probe”
- its source: a database for *BEID* or a platform for *ProbeID*
- the organism to which it refers

A *BEType* can have several *BEType* products but can be the product of at most one *BEType*. This constraint allows the unambiguous identification of the most relevant path to convert identifiers from one scope to another and is fulfilled by the current data model: peptides are only produced from transcripts, which are only produced from genes, which can also code for objects.

*BEID* identifying the same biological entity are related through three different kinds of relationship according to the information available in the source databases, and to the decision made by the database administrator about how to use them. Two *BEID* which *corresponds_to* each other both *identify* the same biological entity. A *BEID* which *is_associated_to* or which *is_replaced_by* another *BEID* does not directly identify any biological entity: the link is always indirect through one or several other *BEID*. Therefore, by design a *BEID* which *is_associated_to* or which *is_replaced_by* another *BEID* can be related to several different biological entities. It is not the case for other *BEID* which identify one and only one biological entity. Relationships *corresponds_to* and *is_associated_to* are used to map identifiers from different databases with different transitivity properties (as explained below), whereas *is_replaced_by* relationships are used to map deprecated identifiers from former versions of the same database. This set of possible relationships allows the indirect mapping of different identifiers not necessarily provided by any integrated resource.

### Implementation

In order to efficiently leverage an indirect path through these different relationships, the data model has been implemented in a Neo4j ^®^ graph database ( Neo4j inc, 2017).

Two R ( R Core Team, 2017) packages have been developed to feed and query the database. The first one, neo2R, provides low level functions to interact with Neo4j ^®^ . The second R package, BED, provides functions to feed and query the BED Neo4j ^®^ graph database according to the data model described above.

### Feeding the database

Many functions are provided within the package to build a tailored BED database instance. These functions are not exported in order not to mislead the user when querying the database (which is the expected most frequent usage of the system). An R markdown document showing how to build a BED database instance for human, mouse and rat organisms is provided within the package. It can be adapted to other organisms or needs.

Briefly, these functions can be divided according to three main levels:

- The lowest level function is the bedImport function which loads a table in the Neo4j ^®^ database according to a Cypher ^®^ query.
- Functions of the second level allow loading identifiers and relationships tables ensuring the integrity of the data model:
  – loadBE, loadProbes, loadOrganisms, loadBeAttribute, loadBEsymbols, loadBENames and loadBEVersion are used to load information about BEID and ProbeID.
  – loadHistory, loadCorrespondsTo, loadIsAssociatedTo, loadIsExpressedAs, loadIsTranslatedIn, loadCodesFor and loadIsHomologOf are used to load relationships existing between BEID and ProbeID.
- Highest level functions are helpers for loading information provided by some public resources in different specific formats. The following resources are currently supported but other resources can be managed by the user by calling the functions mentioned above:
  – Ensembl ( Zerbino *et al.*, 2018): getEnsemblGeneIds, getEnsemblTranscriptIds, getEnsemblPeptideIds
  – NCBI ( NCBI Resource Coordinators, 2017): loadNcbiTax, getNcbiGeneTransPep, loadNCBIEntrezGOFunctions
  – Uniprot ( The UniProt Consortium, 2017): getUniprot
  – Clarivate Analytics MetaBase ^®^ ( Clarivate Analytics, 2017): loadMBObjects

### Available database instance

An instance of the BED database (UCB-Human), built using the script provided in the BED R package, is available in a Docker ^®^ image ( Docker inc, 2017) available here: https://hub.docker.com/r/patzaw/bed-ucb-human/ (tag 2018-04-30). This instance is focused on *Homo sapiens*, *Mus musculus* and *Rattus norvegicus* organisms and it has been built from these resources:

- Ensembl ( Zerbino *et al.*, 2018)
- NCBI ( NCBI Resource Coordinators, 2017)
- Uniprot ( The UniProt Consortium, 2017)
- biomaRt ( Durinck *et al.*, 2009)
- GEOquery ( Davis & Meltzer, 2007)
- Clarivate Analytics MetaBase ^®^ ( Clarivate Analytics, 2017)

The results and the use cases described below were obtained and executed using this instance of the BED database.

The numbers of BEID available in this BED database instance and which can be mapped to each other are shown in Table 1. In total, 3,559,720 BEID are available in this BED instance. This number includes deprecated identifiers without successor and which therefore cannot be mapped to any other identifier. All the genomics platforms included in this BED database instance are shown in Table 2. They provide mapping to BEID from 354,205 ProbeID in total.

**Table 1.** Numbers of BEID available in the BED UCB-Human database instance. Numbers have been split according to the BEType and the organism. Only BEID which can be mapped to each other are taken into account (i.e. excluding deprecated identifiers without successor).

BE	Organism	Database	BEID	URL
Gene	Homo sapiens	MIM_GENE	17,215	http://www.omim.org
Gene	Homo sapiens	miRBase	1,881	http://www.mirbase.org
Gene	Homo sapiens	UniGene	29,237	https://www.ncbi.nlm.nih.gov
Gene	Homo sapiens	Ens_gene	69,056	http://www.ensembl.org
Gene	Homo sapiens	HGNC	41,233	http://www.genenames.org
Gene	Homo sapiens	EntrezGene	81,684	https://www.ncbi.nlm.nih.gov
Gene	Homo sapiens	Vega_gene	19,141	http://vega.sanger.ac.uk
Gene	Homo sapiens	MetaBase_gene	23,356	https://portal.genego.com
Gene	Mus musculus	miRBase	1,193	http://www.mirbase.org
Gene	Mus musculus	UniGene	29,826	https://www.ncbi.nlm.nih.gov
Gene	Mus musculus	Ens_gene	57,601	http://www.ensembl.org
Gene	Mus musculus	MGI	80,387	http://www.informatics.jax.org
Gene	Mus musculus	EntrezGene	103,570	https://www.ncbi.nlm.nih.gov
Gene	Mus musculus	Vega_gene	18,163	http://vega.sanger.ac.uk
Gene	Mus musculus	MetaBase_gene	20,628	https://portal.genego.com
Gene	Rattus norvegicus	miRBase	495	http://www.mirbase.org
Gene	Rattus norvegicus	UniGene	18,570	https://www.ncbi.nlm.nih.gov
Gene	Rattus norvegicus	Ens_gene	34,963	http://www.ensembl.org
Gene	Rattus norvegicus	RGD	46,973	https://rgd.mcw.edu
Gene	Rattus norvegicus	EntrezGene	57,026	https://www.ncbi.nlm.nih.gov
Gene	Rattus norvegicus	Vega_gene	1,146	http://vega.sanger.ac.uk
Gene	Rattus norvegicus	MetaBase_gene	17,505	https://portal.genego.com
Transcript	Homo sapiens	Ens_transcript	233,600	http://www.ensembl.org
Transcript	Homo sapiens	Vega_transcript	34,302	http://vega.sanger.ac.uk
Transcript	Homo sapiens	RefSeq	175,183	https://www.ncbi.nlm.nih.gov
Transcript	Mus musculus	Ens_transcript	139,040	http://www.ensembl.org
Transcript	Mus musculus	Vega_transcript	25,704	http://vega.sanger.ac.uk
Transcript	Mus musculus	RefSeq	114,509	https://www.ncbi.nlm.nih.gov
Transcript	Rattus norvegicus	Ens_transcript	42,393	http://www.ensembl.org
Transcript	Rattus norvegicus	Vega_transcript	1,271	http://vega.sanger.ac.uk
Transcript	Rattus norvegicus	RefSeq	97,882	https://www.ncbi.nlm.nih.gov
Peptide	Homo sapiens	Ens_translation	112,939	http://www.ensembl.org
Peptide	Homo sapiens	Vega_translation	35,332	http://vega.sanger.ac.uk
Peptide	Homo sapiens	RefSeq_peptide	121,813	https://www.ncbi.nlm.nih.gov
Peptide	Homo sapiens	Uniprot	233,935	http://www.uniprot.org
Peptide	Mus musculus	Ens_translation	66,352	http://www.ensembl.org
Peptide	Mus musculus	Vega_translation	26,386	http://vega.sanger.ac.uk
Peptide	Mus musculus	RefSeq_peptide	81,089	https://www.ncbi.nlm.nih.gov
Peptide	Mus musculus	Uniprot	115,377	http://www.uniprot.org
Peptide	Rattus norvegicus	Ens_translation	30,245	http://www.ensembl.org
Peptide	Rattus norvegicus	Vega_translation	1,260	http://vega.sanger.ac.uk
Peptide	Rattus norvegicus	RefSeq_peptide	68,777	https://www.ncbi.nlm.nih.gov
Peptide	Rattus norvegicus	Uniprot	40,789	http://www.uniprot.org
Object	Homo sapiens	MetaBase_object	24,727	https://portal.genego.com
Object	Homo sapiens	GO_function	4,130	http://amigo.geneontology.org
Object	Mus musculus	MetaBase_object	22,000	https://portal.genego.com
Object	Mus musculus	GO_function	4,094	http://amigo.geneontology.org
Object	Rattus norvegicus	MetaBase_object	18,648	https://portal.genego.com
Object	Rattus norvegicus	GO_function	4,060	http://amigo.geneontology.org

**Table 2.** Genomics platforms available in the BED UCB-Human database instance.

Name	Description	BE
GPL6101	Illumina ratRef-12 v1.0 expression beadchip	Gene
GPL6947	Illumina HumanHT-12 V3.0 expression beadchip	Gene
GPL10558	Illumina HumanHT-12 V4.0 expression beadchip	Gene
GPL1355	[Rat230_2] Affymetrix Rat Genome 230 2.0 Array	Gene
GPL1261	[Mouse430_2] Affymetrix Mouse Genome 430 2.0 Array	Gene
GPL96	[HG-U133A] Affymetrix Human Genome U133A Array	Gene
GPL13158	[HT_HG-U133_Plus_PM] Affymetrix HT HG-U133+ PM Array Plate	Gene
GPL571	[HG-U133A_2] Affymetrix Human Genome U133A 2.0 Array	Gene
GPL570	[HG-U133_Plus_2] Affymetrix Human Genome U133 Plus 2.0 Array	Gene
GPL6480	Agilent-014850 Whole Human Genome Microarray 4x44K G4112F	Gene
GPL6885	Illumina MouseRef-8 v2.0 expression beadchip	Transcript

### Transitivity management

In the context of mapping identifiers, transitivity is the inference of a cross-reference between A and C based on existing cross-references between A and B and between B and C. Depending on how biological entities are defined, transitivity is desirable or not. In BED the transitivity mechanism is managed by the two following relationships: *corresponds_to* and *is_associated_to*. On one hand, the *corresponds_to* relationships make the mapping transitive since two BEIDs which are connected through this kind of relationship are considered to *identify* the same BE. On the other hand, a BEID which *is_associated_to* another one does not automatically *identify* the same BE making this kind of relationship not available for transitive mappings. When the BED database is fed, the user chooses which relationship should be of type *corresponds_to* or of type *is_associated_to*. For example, in the instance described above, cross-references provided by Ensembl from Ensembl gene identifiers to Entrez, HGNC and Vega gene identifiers are considered as *corresponds_to* relationships whereas cross-references to miRbase, Unigene and OMIM are considered as *is_associated_to* relationships.

### Querying the database

The *BED* R package provides functions to connect to the Neo4j ^®^ database ( connectToBed) and to directly query it ( bedCall). Beside these two functions, others of higher level are provided to explore available data, to manage identifiers and to convert them from one scope to another.

Different functions can be used to explore the data model by listing the type of BE ( listBe) or describing their relationships ( firstCommonUpstreamBe). Available organisms, databases and platforms can also be retrieved using listOrganisms, listBeIdSources and listPlatforms functions.

All BEID from a specific scope can be obtained with the getBeIds function. A specific BEID and its relationships with others can be graphically explored with the exploreBe function. The use and the results of these two functions are exemplified in Results. Also, the functions guessIdOrigin and checkBeIds are used to guess and to check the scope of any list of identifiers. This set of functions is completed by the getBeIdSymbols, getBeIdNames, getGeneDescription and getBeIdDescription functions which provide different ways to annotate identifiers taking advantage of information related to connected BEID. Other functions, searchId and getRelevantIds, are also provided to seek relevant identifiers for a specific BE. These functions are used by a shiny ( Chang *et al.*, 2017) gadget ( findBe) providing an interactive dictionary of BEID which is also made available as an Rstudio add-in ( Allaire *et al.*, 2017; Cheng, 2016).

As described above, the BED data model has been built to fulfill molecular biology processes in order to ensure the biological relevance of identifier mappings. The *is_expressed_as* and *is_translated_in* relationships correspond to the transcription and translation processes respectively whereas *codes_for* is a fuzzy relationship allowing the mapping of genes on objects not necessarily corresponding to the same kind of biological molecules. These processes are described in different databases with different levels of granularity. For example, Ensembl ( Zerbino *et al.*, 2018) provides possible transcripts for each gene specifying which of them is canonical. The getDirectProduct and getDirectOrigin functions allow the user to retrieve direct products or direct origins of such molecular biology processes.

The automatic conversion of identifiers from one scope to another is handled by the convBeIds function. This conversion process can be applied directly on lists of identifiers ( convBeIdLists) or on data frames with an identifier column ( convDfBeIds). Because such conversion can be intricate, the exploreConvPath function is provided to display the shortest relevant paths between two identifiers. These functions are also exemplified in Results and Use case.

Converting thousands of identifiers can take some time (generally a few seconds). Also, such conversions are often recurrent and redundant. In order to improve the performance for such recurrent and redundant queries, a cache system has been implemented. The first time, the query is run on Neo4j ^®^ for all the relevant ID related to user input and the result is saved in a local file. Next time similar queries are requested, the system does not call Neo4j ^®^ but loads the cached results and filters it according to user input. By default, the cache is flushed when the system detects inconsistencies with the BED database. If needed, it can also be manually flushed by using the lsBedCache and clearBedCache functions.

### Operation

Minimal system requirements for running BED and neo2R R packages:

- **R** ≥ 3.4
- **Operating system**: Linux, macOS, Windows
- **Memory** ≥ 4GB RAM

The graph database has been implemented with Neo4j ^®^ version 3 ( Neo4j inc, 2017). The BED R package depends on the following packages available in the Comprehensive R Archive Network ( CRAN):

- *visNetwork* ( Almende *et al.*, 2017)
- *dplyr* ( Wickham *et al.*, 2017)
- *htmltools* ( RStudio inc, 2017)
- *DT* ( Xie, 2016)
- *shiny* ( Chang *et al.*, 2017)
- *miniUI* ( Cheng, 2016)
- *rstudioapi* ( Allaire *et al.*, 2017)

## Results

The results below exemplify how BED efficiently tackle the three challenges described in the introduction. (1) The way identifiers are managed allow the mapping of deprecated identifiers. (2) The identifier conversion process takes advantage of the transitivity mechanism described in Methods to improve its completeness. (3) Mapping rules between different types of biological entities (BE) allow the correct and automatic conversion of identifiers. Finally, BED run time has been compared to three other tools in different contexts. These results were obtained using the instance of the BED database described in Methods and which is available in Docker Hub: https://hub.docker.com/r/patzaw/bed-ucb-human/ (tag 2018-04-30).

### Management of identifiers

Identifiers (ID) in BED can identify a biological entity (BEID) or a probe (ProbeID). The getBeIds function returns all ID from a specific scope. As described in Methods a scope is defined by its type (the type of BE or “Probe”), its source (a database for BEID or a platform for ProbeID) and the organism to which it refers. For example, the following code returns all the Ensembl identifiers of human genes.



beids <- getBeIds (
    
	be= "Gene" , source= "Ens_gene" , organism= "human" ,
    restricted= FALSE

)

head (beids)
                    




                        ##                    id preferred  Gene db.version db.deprecated
## 83452 ENSG00000276626      TRUE 65397         92         FALSE
## 83453 ENSG00000199595      TRUE 65401         92         FALSE
## 83454 ENSG00000201381     FALSE 65401         54      20090519
## 83468 ENSG00000200605      TRUE 65406         92         FALSE
## 83469 ENSG00000206757     FALSE 65406         54      20090519
## 83455 ENSG00000207395      TRUE 65407         92         FALSE
                    


**Figure 2.**
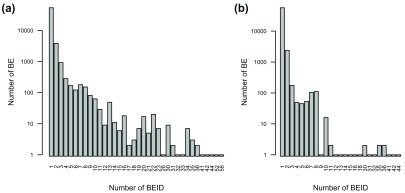
Barplots showing the number (log scale) of gene BE identified by one or more Ensembl gene BEID. **a**) All Ensembl gene ID. **b**) Current Ensembl gene ID (version 92).

The *id* column corresponds to the BEID from the source of interest. The column named according to the type of BE (in this case *Gene*) corresponds to the internal identifiers of the related BE. This internal identifier is not a stable reference that can be used as such. Nevertheless, it is useful to identify BEID identifying the same BE. In the example above, even if most Gene BE are identified by only one Ensembl gene BEID, many of them are identified by two or more (6,031/59,901 ≈ 10%); 277 BE are even identified by more than 10 Ensembl BEID ( Figure 2a). In this case, most of these redundancies come from deprecated BEID from former versions of the Ensembl database (version in use here: 92) and can be excluded by setting the restricted parameter to TRUE when calling the getBeIds function ( Figure 2b). However, many BE are still identified by two or more current Ensembl BEID (2,944/59,901 ≈ 5%). This result comes from the way the BED database is constructed: When two BEID from the same resource correspond to the same BEID in another resource ( *correspond_to* relationship in the data model), all these BEID are considered to identify the same BE.

**Figure 3.**
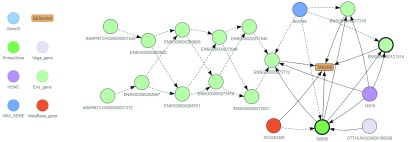
BED relationships between all the different identifiers of the human TAS2R8 gene recorded in the database. BEID are shown as circle and gene symbol in the rounded box. The color legend is shown to the left of the figure. BEID surrounded in bold correspond to *preferred* identifiers. Solid arrows represent *correspond_to* (between two nodes of identical shape) and *is_known_as* (between two nodes of different shapes) relationships. Dotted arrows represent *is_replaced_by* (between two nodes of identical color) and *is_associated_to* (between two nodes of different colors) relationships. This graph has been drawn with the exploreBe function.

A complex example of such mapping is shown in Figure 3 mapping all the BEID of the human TAS2R8 gene which codes for a protein of the family of candidate taste receptors. There are three identifiers corresponding to this gene symbol in Ensembl. All these three BEID correspond to the same Entrez gene and the same HGNC identifiers. All these BEID are thus considered to identify the same gene. It turns out that the three Ensembl BEID correspond to the same gene mapped on different sequence versions of the chromosome 12: the canonical (ENSG00000121314), CHR_HSCHR12_2_CTG2 (ENSG00000272712) and CHR_HSCHR12_3_CTG2 (ENSG00000277316). This information provided by Ensembl is encoded in the *seq_region* attribute for each Ensembl BEID (see data model) and is used to define *preferred* BEID which are mapped on canonical versions of chromosome sequences. The ENSG00000272712 identifier shows also a complex history in former Ensembl versions.

### Converting gene identifiers

The main goal of BED is to convert identifiers from one scope to another easily, rapidly and with high completeness. It has been thought in order to allow recurring comparisons to each other of many lists of BEID from various origins.

A simple example regarding the conversion of human Ensembl gene to human Entrez gene identifiers is shown below and discussed hereafter. By setting the restricted parameter to TRUE the converted BEID are restricted to current - non-deprecated - version of Entrez gene identifiers. Nevertheless, all the input BEID are taken into account, current and deprecated ones.



bedConv <- convBeIds (
   
	ids= beids $ id, from= "Gene" , from.source= "Ens_gene" , from.org= "human" ,
   
    to.source= "EntrezGene" , restricted= TRUE

)
                    


Among all the 69,056 human Ensembl gene identifiers available in the database, 22,056 (32%) were not converted to any human Entrez gene identifier: 21,416 (33%) of the 65,256 non-deprecated and 640 (17%) of the 3,800 deprecated identifiers.

In order to assess the improvement of completeness achieved by BED, we compared it to three other tools: biomaRt ( Durinck *et al.*, 2009; Kinsella *et al.*, 2011), mygene ( Mark *et al.*, 2014; Wu *et al.*, 2013), and gProfileR ( Reimand *et al.*, 2016a; Reimand *et al.*, 2016b). All these tools were used on May 03, 2018 to perform the same conversion task. At that time, biomaRt was based on the Ensembl 92 release (as the BED database instance), mygene on release 91 and gProfileR on release 90.

**Figure 4.**
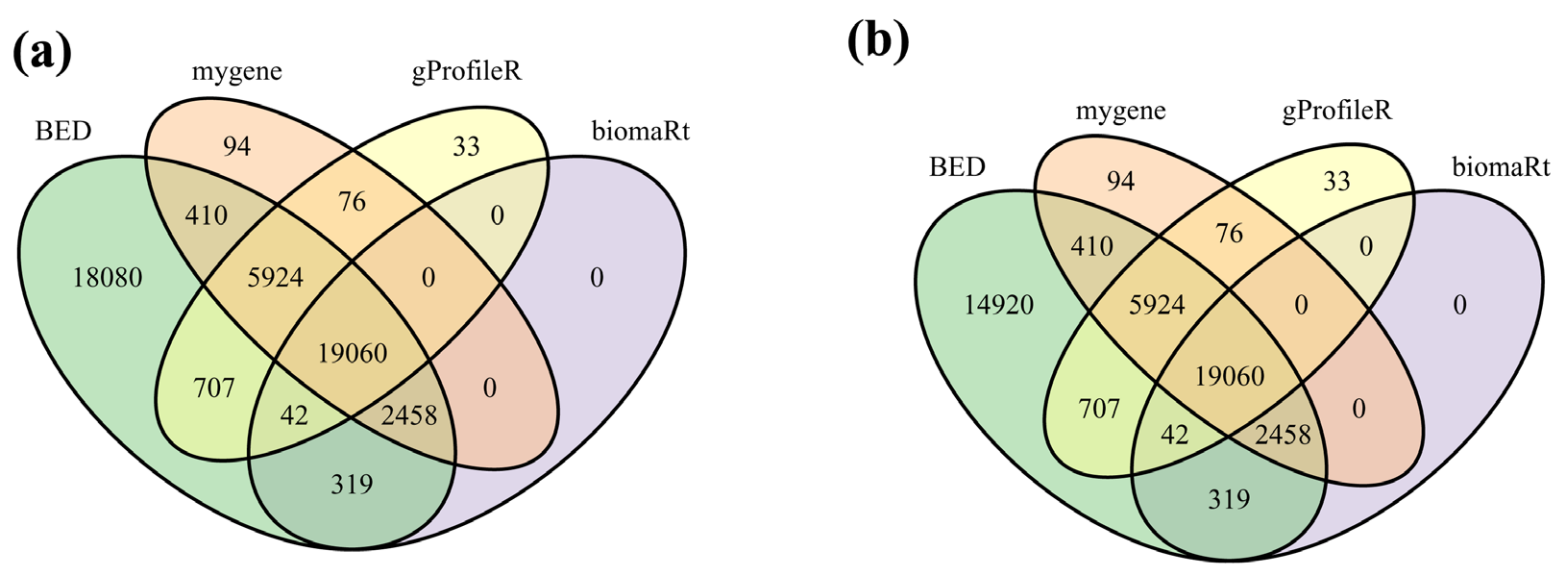
Venn diagrams showing the number of human Ensembl gene identifiers mapped to at least one human Entrez gene identifier by the different tested tools when focusing ( **a**) on all 69,056 or ( **b**) on current 65,256 BEID (Ensembl 92 release).

The numbers of human Ensembl gene identifiers successfully converted by each method are compared in Figure 4. Mappings returned only by gProfileR or by mygene (33 + 94 + 76) are available in releases 90 and 91 of Ensembl respectively but not in release 92. They probably correspond to deprecated cross-references. Conversely, mappings returned by both BED and biomaRt but neither by gProfileR nor mygene (319) are available in release 92 of Ensembl but not in releases 91 or 90. All the gene identifiers successfully converted by biomaRt were also converted by BED. However, BED was able to map at least 18,080 more identifiers than all the other tools ( Figure 4a). A few of these mappings (3,160) are explained by the fact that BED is the only tool mapping deprecated identifiers to current versions. Nevertheless, even when focusing on the mapping of current versions of Ensembl identifiers, BED was able to map 14,920 more identifiers than all the other tools ( Figure 4b). A few of these mappings (683) are directly provided by the NCBI. But most of them (14,237) are inferred from a mapping of the Ensembl and Entrez gene identifiers to the same HGNC ( Gray *et al.*, 2015) identifier.

We assessed the validity of the mappings by comparing the location on the human genome (GRCh38) of Ensembl gene identifiers as reported by Ensembl and the location of the corresponding Entrez gene identifiers as reported by the NCBI. If the mapping between two identifiers is correct their location on the genome should be identical or highly similar (only gene identifiers located on canonical versions of chromosome sequences were considered for this comparison). We compared the following mapping results:

- The “Reference” mappings provided by biomaRt for identifiers successfully converted by all the tools were considered as the reference (19,166 regard genes on canonical versions of chromosomes).
- The “Former” mappings provided by mygene for identifiers successfully converted by mygene but neither by biomaRt nor BED correspond to mappings only available in the former version (91) of Ensembl (67 regard genes on canonical versions of chromosomes).
- The “BED” mappings provided by BED for identifiers successfully converted by BED but not by any of the three other tools correspond to the mappings achieved mostly thanks to transitivity through HGNC identifiers (12,913 regard genes on canonical versions of chromosomes).

**Figure 5.**
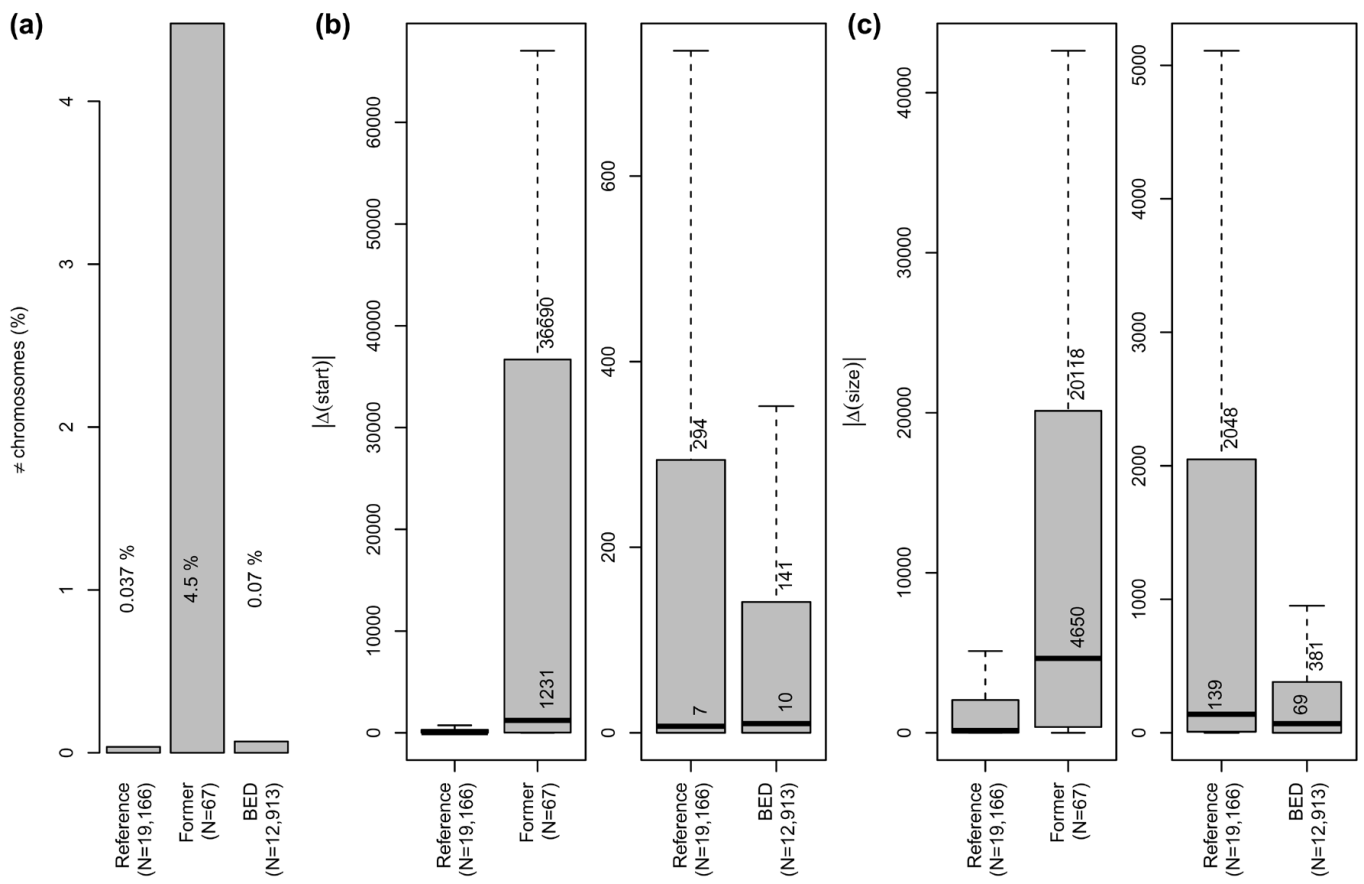
Comparison of gene locations provided by Ensembl for Ensembl identifiers and by the NCBI for the cross-referenced Entrez identifiers. “Reference” corresponds to mappings provided by biomaRt for identifiers successfully converted by all the tools. “Former” corresponds to mappings provided by mygene for identifiers successfully converted by mygene but neither by biomaRt nor BED. “BED” corresponds to mappings provided by BED for identifiers successfully converted by BED but not by any of the three other tools. Numbers of considered mappings (N) in each group are indicated (see text for details). **a**) Proportion of cross-referenced identifiers located on different chromosomes. **b**) Absolute distance (base pairs) between gene start positions for cross-referenced identifiers located on the same chromosome. **c**) Absolute difference (base pairs) between gene sizes for cross-referenced identifiers located on the same chromosome. Median and third quartile values are indicated. Outliers are not shown. “BED” and “Former” results are compared to Reference on different boxplots because of scale shift.

As shown in Figure 5a, the proportion of former cross-referenced identifiers (“Former”) located on different chromosomes is much higher than for the reference (“Reference”). The proportion of BED cross-referenced identifiers (“BED”) located on different chromosomes is higher than for “Reference” but still low. Figures 5.a and b show that inconsistencies in gene start location and in gene size are much more important in “Former” mappings than in “Reference” mappings. Such difference is not observed between the “Reference” mappings and the additional mappings provided by BED. These results show that the general quality of “Former” mappings is lower than the “Reference”, which is in agreement with their deprecation. More interestingly, additional mappings provided by BED are of similar quality to the “Reference” making their use as valuable.

As shown above, additional mappings between Ensembl and Entrez gene identifiers inferred thanks to the use of HGNC cross-references by BED are many and of good quality. However, this transitivity mechanism is not always desirable depending on how different resources define biological entities. It is especially true for gene which is an unstable concept as described by Gerstein *et al.* (2007). For example, in Entrez the Hs.103110 UniGene identifier is mapped to 5465 and to 150383 Entrez gene identifiers which correspond to two different genes in Entrez (PPARA and CDPF1) but also in Ensembl ( ENSG00000186951 and ENSG00000205643) and in HGNC ( 9232 and 33710). These two genes are located closely on the same chromosome but at different positions and on different directions. The same feature has been observed for many UniGene identifiers which makes UniGene unsuitable for transitivity mappings between gene identifiers. Therefore, in this instance of the BED database, as described in Methods, cross-references between Ensembl and Unigene gene identifiers are considered as *is_associated_to* relationships avoiding transitivity.

### Mapping rules

Beside cross-referencing identifiers of identical type of BE, BED uses the biological relationship between genes, transcript and peptides to convert identifiers across different type of BE. For example, when converting peptides identifiers from the same species it uses only mapping done at the peptide level and does not use mapping to transcripts and genes. This strategy seems to be applied by biomaRt but not by mygene nor by gProfileR which map, for example, one Uniprot identifier to all the Ensembl peptide identifier coded by the same gene. For example, the A6NI28 Uniprot identifier is unambiguously mapped to the ENSP00000298815 Ensembl peptide identifier by BED and biomaRt but is wrongly mapped to three additional Ensembl peptide identifiers by mygene and gProfileR ( ENSP00000431776, ENSP00000434304 and ENSP00000435961) which are encoded by the same gene ( ENSG00000165895).

Furthermore, in biomaRt, mygene and gProfileR, mapping of BEIDs that are not genes from two different organisms using orthologs information requires at least two steps: one to find the ortholog genes and the other to find the relevant BEID. These two steps are integrated and transparent in BED.

In general, thanks to the BED data model and to mapping rules, identifier conversions from one scope to another are biologically relevant and automatic as exemplified in the use case described below.

**Figure 6.**
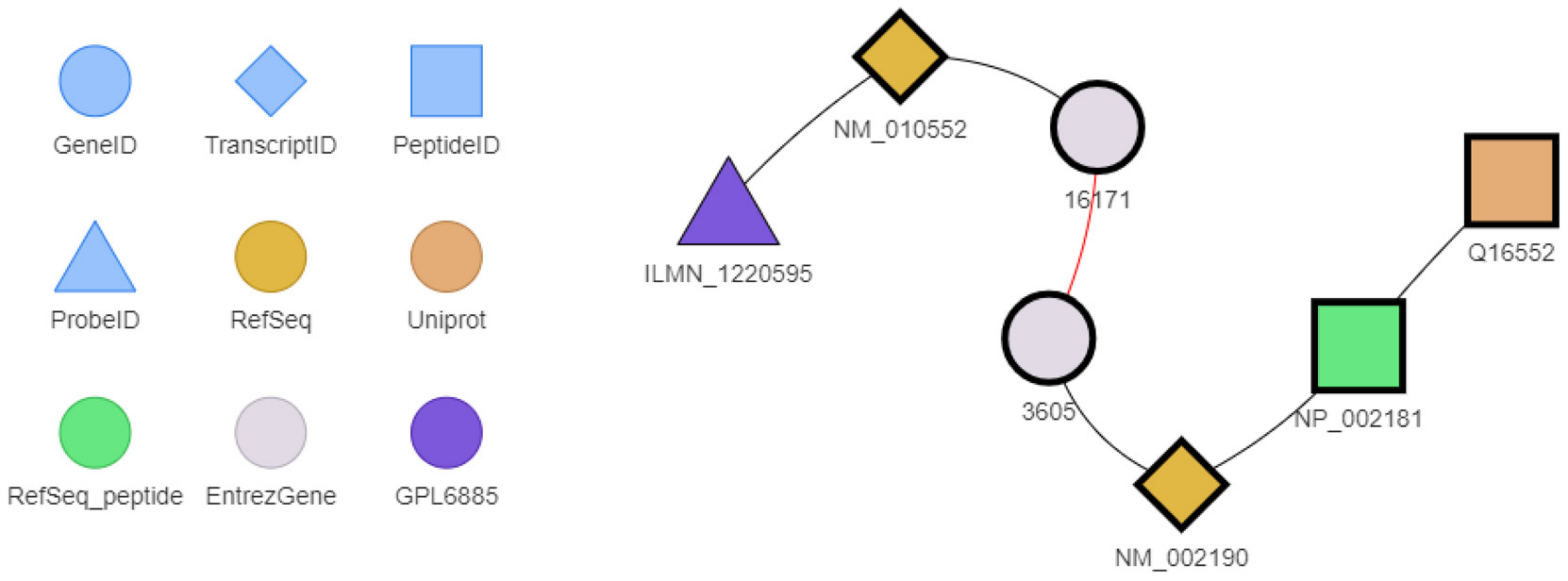
BED conversion shortest path between the ILMN_1220595 probe identifier targeting a transcript of the mouse Il17a gene and the Uniprot Q16552 identifier of the human IL17 protein. The legend is shown to the left of the figure. The red edge represents the *is_homolog_of* relationship. BEID surrounded in bold correspond to *preferred* identifiers. This graph has been drawn with the exploreConvPath function.

Because all these indirect mappings can be intricate, BED provides a function to show the shortest relevant paths between two different identifiers ( Figure 6).

### Performance

The time taken by BED to convert different sets of identifiers from one scope to another has been compared to the time taken by biomaRt, mygene and gProfileR. Three different queries have been executed starting from 100 or 20,000 identifiers.

**Figure 7.**
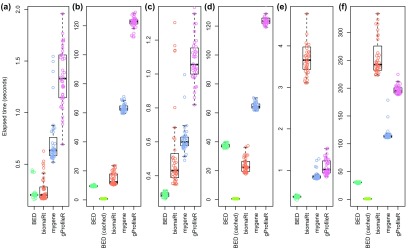
Comparison of the time taken to convert a set of randomly selected BE identifiers. Random selections of 100 or 20,000 identifiers have been executed 40 times before conversion by each considered tools. BED performance was assessed without using its cache system for all the queries and also using its cache system for queries regarding 20,000 identifiers. **a**) Conversion of 100 Ensembl human gene ID into Entrez gene ID. **b**) Conversion of 20,000 Ensembl human gene ID into Entrez gene ID. **c**) Conversion of 100 Uniprot mouse peptide ID into Ensembl transcript ID. **d**) Conversion of 20,000 Uniprot mouse peptide ID into Ensembl transcript ID. **e**) Conversion of 100 Affymetrix probe ID into Ensembl mouse peptide ID. **f**) Conversion of 20,000 Affymetrix probe ID into Ensembl mouse peptide ID.

As shown in Figure 7, BED outperforms mygene and gProfileR in all the tested cases even when not using its cache system. It also outperforms biomaRt for converting Affymetrix probe ID into Ensembl mouse peptide ID ( Figures 7.e and 7.f). Without using its cache system, BED performs equally well as biomaRt for converting Ensembl human gene ID into Entrez gene ID ( Figures 7.a and 7.b). It outperforms biomaRt for converting 100 Uniprot mouse peptide ID into Ensembl transcript ID ( Figures 7.c) but is outperformed by biomaRt when 20,000 identifiers are considered ( Figures 7.d). Nevertheless, when cache is used, BED outperforms all the tools whatever the conversion scenario ( Figures 7.b, 7.d and 7.f). These results show that BED, as a dedicated and locally available tool, is a very efficient option to convert large lists of identifiers on the fly and recurrently.

## Use case: comparing transcriptomics data sets

In this use case, we show how BED facilitates the comparison of data sets relying on identifiers with different scopes. Indeed, the mechanisms implemented in the BED R package make easy the conversion of a set of identifiers from one scope to any other. Thus, the comparison of data from different genome wide experiments is straightforward from a technical point of view.

To exemplify this statement, we show below how to compare differential expression data from three experiments addressing the understanding of Psoriasis mechanisms with different experimental designs and platforms:

- Nair *et al.* (2009) compared gene expression data in lesional and non-lesional skin from Psoriasis patients using Affymetrix GeneChip Human Genome U133 Plus 2.0. Starting from published data (Array-Express ( https://www.ebi.ac.uk/arrayexpress) accession number: E-GEOD-13355) we recomputed differential expression between these two groups of samples.
- Chiricozzi *et al.* (2011) measured transcriptomics responses to IL-17 and TNF- *α* cytokines in human keratinocytes using Illumina HumanHT-12 v3.0 Expression BeadChips. Starting from published data ( E-GEOD-24767) we recomputed differential expression between keratinocytes co-stimulated with both cytokines and controls.
- Swindell *et al.* (2011) compared gene expression data in lesional and non-lesional skin from five psoriasis mouse models using Affymetrix GeneChip Mouse Genome 430 2.0. Starting from published data ( E-GEOD-27628) we recomputed differential expression between affected and non-affected skin samples.

The first rows of these three data tables are shown below.



## E-GEOD-13355: Human skin

head (hsSkin.DE, n= 3 )
                




## 	        logFC 	   P.Value    adj.P.Val
## 41469_at  5.140278 8.384147e-46 4.584033e-41
## 232170_at 5.766759 3.293084e-45 9.002469e-41
## 205863_at 4.715087 5.796620e-45 1.056434e-40
                




## E-GEOD-24767: Human keratinocytes

head (hsKera.DE, n= 3 )
                




## 		   logFC      P.Value    adj.P.Val
## ILMN_2048043 6.297295 2.186104e-14 1.066841e-09
## ILMN_1672295 3.507524 5.823956e-12 1.051466e-07
## ILMN_1680965 6.114791 6.463797e-12 1.051466e-07
                




## E-GEOD-27628: Mouse skin

head (mmSkin.DE, n= 3 )
                




## 	           logFC      P.Value   adj.P.Val
## 1440888_at -1.3929330 3.317833e-08 0.000732987
## 1449319_at -0.9672017 4.105579e-08 0.000732987
## 1422803_at -0.7688354 5.906985e-08 0.000732987
                


The aim of the following commands is to allow the comparison of the three logFC values by converting ProbeID (row names) of one or the other data set. The function guessIdOrigin is used to identify the scope of the different sets of identifiers.



scopes <- data.frame (
   
"Human skin" = unlist ( guessIdOrigin ( rownames (hsSkin.DE), tcLim= 1000 )),
    
	"Human keratinocytes" = unlist ( guessIdOrigin ( rownames (hsKera.DE), tcLim= 1000 )),
   
    "Mouse skin" = unlist ( guessIdOrigin ( rownames (mmSkin.DE), tcLim= 1000 )),
   
    stringsAsFactors= FALSE , check.names= FALSE

)
scopes
                




## 	      Human skin    Human keratinocytes    Mouse skin
## be 		   Probe 	          Probe         Probe
## source     	  GPL570 	       GPL10558       GPL1261
## organism Homo sapiens           Homo sapiens  Mus musculus
                


To compare the two human data sets, the keratinocyte data set can be converted to the same scope of the skin data set using the convDfBeIds function. After conversion, the two data sets can be merged before computing the correlation between logFC values.



## Human skin VS keratinocytes
convHsKera.DE <- 
convDfBeIds (
    df= hsKera.DE,
   
    from= scopes[ "be" , "Human keratinocytes" ],
   
    from.source= scopes[ "source" , "Human keratinocytes" ],
   
    from.org= scopes[ "organism" , "Human keratinocytes" ],
   
    to= scopes[ "be" , "Human skin" ],
   
    to.source= scopes[ "source" , "Human skin" ],
   
    to.org= scopes[ "organism" ,  "Human skin" ],
   
    restricted= TRUE

)

toCompare <- merge (
   
    hsSkin.DE[, "logFC" , drop= FALSE ],
   
    convHsKera.DE[, c( "conv.to" , "logFC" )],
   
    by.x= 0 , by.y= "conv.to"

)

colnames (toCompare) <- c ( "ID" , "Skin" , "Keratinocytes" )

round ( cor (toCompare $ "Skin" , toCompare $ "Keratinocytes" ), 2 )
                




## [1] 0.15
                


A similar procedure can be applied to compare the two skin data sets or to compare mouse skin and human keratinocytes.



## Human skin VS mouse skin
convMmSkin.DE <- 
    convDfBeIds (
   
    df= mmSkin.DE,
   
    from= scopes[ "be", "Mouse skin" ],
   
    from.source= scopes[ "source" , "Mouse  skin" ],
   
    from.org= scopes[" organism" , "Mouse  skin" ],
   
    to= scopes[ "be" , "Human  skin" ],
   
    to.source= scopes[ "source" , "Human  skin" ],
   
    to.org= scopes[ "organism" , "Human  skin" ],
   
    restricted= TRUE

)

toCompare <- merge (
   
    hsSkin.DE[, "logFC" , drop= FALSE ],
   
    convMmSkin.DE[, c ( "conv.to" , "logFC" )],
   
    by.x= 0 , by.y= "conv.to"

)

colnames (toCompare) <- c ( "ID" , "Human" , "Mouse" )

round ( cor (toCompare $ "Human" , toCompare $ "Mouse" ), 2
)
                




## [1] 0.24
                




##  Mouse  skin  VS  human  keratinocytes

conv2HsKera.DE  <- convDfBeIds (
   
    df= hsKera.DE,
   
    from= scopes[ "be" , "Human  keratinocytes" ],
   
    from.source= scopes[ "source" , "Human  keratinocytes" ],
   
    from.org= scopes[ "organism" , "Human  keratinocytes" ],
   
    to= scopes[ "be" , "Mouse  skin" ],
   
    to.source= scopes[ "source" , "Mouse  skin" ],
   
    to.org= scopes[ "organism" , "Mouse  skin" ],
   
    restricted= TRUE

)

toCompare <- merge (
   
    mmSkin.DE[, "logFC" , drop= FALSE ],
   
    conv2HsKera.DE[, c ( "conv.to" , "logFC" )],
   
    by.x= 0 , by.y= "conv.to"

)

colnames (toCompare) <- c ( "ID" , "Mouse skin" , "Human keratinocytes" )

round ( cor (toCompare $ "Mouse skin" , toCompare $ "Human keratinocytes" ), 2 )
                




## [1] 0.07
                


As shown above, converting the identifier scope of transcriptomic data sets is straightforward and quickly executed using BED. It makes the comparison (such as correlation analyses performed in this use case) and the integration of multiple heterogeneous data very easy to achieve.

Additional examples of BED functionalities are provided in the R package vignette.

## Conclusions

The appearance of “omics” technologies, biological knowledge databases and systems biology analytical approaches have opened the possibility to integrate various data sets to get a better understanding of biological processes underlying different complex phenotypes such as diseases. However, this promising interoperability of data sets is largely hampered by the heterogeneity of identifiers used by technical platforms but also those used by knowledge databases to organize information. Comparing and integrating all these data requires the ability to map the identifiers on which each resource relies. Many tools have been developed to achieve this task (e.g. Kinsella *et al.* (2011); Reimand *et al.* (2016a); Wu *et al.* (2013) or van Iersel *et al.* (2010)). However, we identified three challenges generally not addressed by the available tools:

- Information provided by different data sources is not leveraged to increase the completeness of identifier conversion.
- Deprecated identifiers, used in former versions of resources, are not available anymore for conversion in up-to-date versions of mapping tools, damaging the integration of historical data sets.
- The mapping between very different scopes of identifiers is either difficult to automate or not biologically relevant.

BED is a system dedicated to the mapping between identifiers of molecular biological entities. It relies on a graph data model implemented with Neo4j ^®^ and on rules coded in an R package. BED leverages mapping information provided by different resources in order to increase the mapping completeness between each of them. It also allows the mapping of deprecated identifiers. Rules are used to automatically convert identifiers from one scope to another using the most appropriate path.

The intent of BED is to be tailored to specific needs and beside functions for querying the system the BED R package provides functions to build custom instances of the database, including internal or proprietary resources for example. Database instances can be locally installed or shared across a community. This design combined with a cache system makes BED efficient for converting large lists of identifiers from and to a large variety of scopes. Because of our research field we provide an instance focused on human, mouse and rat organisms. This database instance can be directly used in relevant projects but it can also be enriched depending on user or community needs.

Beside a casual use for analyzing and comparing data in the frame of a research project, BED can be advantageously employed by systems dealing with biological and molecular information from heterogeneous sources. Indeed, various pieces of knowledge can be efficiently managed in their original scope (e.g. gene ID for genomics or epigenomics data, transcript ID for transcriptomics data and protein ID for proteomics data). Thanks to the automatic and fast conversion of identifiers all these data can then be compared to each other or to any user input. We do not provide BED API for other languages than R but we expect that publishing the source code under an open source license (GPL-3) will encourage other developers to extend or improve it according to their needs and their expertise.

## Software availability

Latest source code is available at:

https://github.com/patzaw/BED

https://github.com/patzaw/neo2R

Archived source code as at time of publication:

http://doi.org/10.5281/zenodo.1244150 ( Godard, 2018a)

http://doi.org/10.5281/zenodo.1167670 ( Godard, 2018b)

Archived database docker image as at time of publication:

http://doi.org/10.5281/zenodo.1311362 ( Godard, 2018c)

Software is available to use under a GPL-3 license
