# Supplementary material for: BED: a Biological Entity Dictionary based on a graph data model
Source: F1000Res. 2018 Jul 19;7:195. Originally published 2018 Feb 15. [Version 3] doi: 10.12688/f1000research.13925.3 (PMC6039941; doi:10.12688/f1000research.13925.3)
Supplement: Peer review history

# Peer review: Referee response for version 3

**DOI:** 10.5256/f1000research.17046.r36266

**Published:** 2018-07-20

**Authors:** T. Ian Simpson

**Affiliations:** 1 School of Informatics , University of Edinburgh, Edinburgh, UK, UK

Happy with these additional minor changes.

I have read this submission. I believe that I have an appropriate level of expertise to confirm that it is of an acceptable scientific standard.

# Peer review: Referee response for version 2

**DOI:** 10.5256/f1000research.16282.r34080

**Published:** 2018-07-03

**Authors:** Martina M. Summer-Kutmon, Denise Slenter

**Affiliations:** 1 Department of Bioinformatics - BiGCaT, Maastricht University, Maastricht, Netherlands

We would like to thank the authors for addressing our comments and incorporating them nicely in the new manuscript version. Additionally, they also added a lot more details and figures to many of the sections, which further clarify the approach and relevance of BED. We also really appreciate the added results and use case!

 We only have some small remarks that might be nice to address:

- Methods / Data model → deprecated identifiers are one of the challenges addressed by BED, but it is not discussed how those are represented in the data model.
- Methods / Available database instance → it would be nice to provide a DOI for the specific docker instance release.
- Results / Figure 6 → legend should now say “The red edge” instead of “The red arrow”.
- Results / Figure 7 → we believe legend for figure f should be “Conversion of **20,000**​ Affymetrix probe ID into Ensembl mouse peptide ID”

We have read this submission. We believe that we have an appropriate level of expertise to confirm that it is of an acceptable scientific standard.

# Peer review: Referee response for version 2

**DOI:** 10.5256/f1000research.16282.r34079

**Published:** 2018-06-13

**Authors:** T. Ian Simpson

**Affiliations:** 1 School of Informatics , University of Edinburgh, Edinburgh, UK, UK

Many thanks to the authors for making changes to the manuscript in response to my and the other reviewers comments. It is particularly nice to see the data for validation  (Fig.5) and speed of execution (Fig.7) added. I am happy to recommend the article for indexing in this amended form.

I have read this submission. I believe that I have an appropriate level of expertise to confirm that it is of an acceptable scientific standard.

# Peer review: Referee response for version 1

**DOI:** 10.5256/f1000research.15138.r31928

**Published:** 2018-03-26

**Authors:** T. Ian Simpson

**Affiliations:** 1 School of Informatics , University of Edinburgh, Edinburgh, UK, UK

In this article the authors present BED, a biological entity database implemented as a Neo4J labelled property graph. In addition, they provide two R-packages (BED & neo4J) for the construction and query of such graphs that adhere to their data model. These packages include utility functions to facilitate graph construction from a range of commonly used and publicly available data sources. The software and database are well documented and available through GitHub and Docker (as a Docker image) respectively and proved straight forward to install and run.

 There are several elements of the current manuscript that warrant commentary:

1. **Motivation/Rationale**. The authors have correctly identified an important problem with the integration of biological data that has been addressed before, not least by the resources/tools mentioned in the manuscript (Biomart, my gene, g:Profiler amongst others). They have chosen a particularly good approach (labelled property graphs) to build the data architecture to address such a problem and one that has recently been used to great effect by the EMBL-EBI Reactome team to model data related to biological pathways. Currently this manuscript somewhat undersells the potential for the tools that have been developed. Whilst allusion is made at various points to the fact that the software developed could be used by others to develop custom resources very little is presented as to the suitability of their approach for such. The "Abstract" states that existing resources "cannot be customised and optimised for any specific use" which is not correct and should be removed or re-worded to clarify the author's meaning. Whilst the implementation presented here is focussed primarily on gene level mappings it should be made clear throughout the manuscript that the general approach used could be (and indeed has been, see citation) used in other really quite different biological data modelling scenarios.
2. **Introduction**. The issue of "transitivity" is raised here, this is a complex issue for many biological data types that are far removed from the rigid structures of ontologies that commonly enforce it by definition. The meaning of "transitivity" in the context used here is not clear and warrants further explanation. This is particularly important later in the article where decisions are being made about inferring mappings where they don't exist in the data. Some such inferences are entirely logical (e.g. using HGNC ids two link gene_ids between two resources that don't map directly to each other) but others are far more complex (e.g. mapping between species). The inclusion of deprecated identifiers is excellent and will help to close a notable gap in many existing resources for which mapping older data into more recent datasets can be extremely time consuming and frustrating. The authors comment on "mapping between different scopes" is unclear and should be clarified.
3. **Methods**. The sections "Feeding the Database" and "Querying the Database" are very brief and would benefit from much more detail about the functionality of the database creation and query system. Whilst these are covered in detail in the various pieces of documentation (including some very nice working examples) there is not enough in the manuscript itself to allow the reader to assess the available functionality.
4. **Use Cases**.

- There appears to be a discrepancy in gene counts from the Ensembl examples used in this section; the first example calls human Ensembl genes and returns 59,515 genes the second states the total number of human Ensembl genes to be 68,460.
- Figure 3. illustrates a relationship graph including deprecated BEIDs. Whilst *is_replaced_by* is clear, it is not clear (or defined anywhere) what the meaning of *is_associated_to* is and how that differs from *corresponds_to*.  This should be clarified in the text.
- The sentence "The function *guessIdOrigin*..." appears out of place, unconnected to the surrounding text.
- The statement "Five identifiers were only..." and the following sentence should be combined and re-worded so that the explanation as to why 5 BEIDs were uniquely found by gProfiler is clearer.
- No validation or commentary has been presented to test the efficacy of inferences made by the query system. I would like to have seen an attempt made to check the veracity of mappings made in this way especially when the majority (c.80%) of extra Ensmembl->EntrezID mappings recovered via BED were inferred.
- A "rough approximation" of timings for queries within BED and across other systems is not particularly informative. It would have been straightforward to automate a sampling approach to generate a mean response time (and a variance) to a defined set of query sizes/complexities to give the user a better understanding of how variable these response times are between the systems in practice. In addition, it would have been nice to see some analysis/discussion about the "scalability" of the system as this is likely to be of particular interest to end-users considering a similar modelling approach in other domains.
- Figure5. The meaning of directionality here is not clear. Whilst I can see the benefit for provenance reasons i.e. a mapping from EntrezGene **to** RefSeq it's meaning here is somewhat moot.

The work presented in this manuscript promises to be very useful for researchers wanting to use LPGs for data integration. The implementation and deployment have been very well executed so that they can be readily adopted and modified by end-users.

I have read this submission. I believe that I have an appropriate level of expertise to confirm that it is of an acceptable scientific standard, however I have significant reservations, as outlined above.

## References

[1] FabregatAJupeSMatthewsLSidiropoulosKGillespieMGarapatiPHawRJassalBKorningerFMayBMilacicMRocaCDRothfelsKSevillaCShamovskyVShorserSVarusaiTViteriGWeiserJWuGSteinLHermjakobHD'EustachioP: The Reactome Pathway Knowledgebase. *Nucleic Acids Res*.2018;46(D1) : 10.1093/nar/gkx1132 D649-D655 10.1093/nar/gkx1132 29145629PMC5753187

# Response

**Published:** 2018-04-26

**Authors:** Patrice Godard

**Affiliations:** UCB, Belgium

Thanks for having taken the time to review this article and for your constructive comments that will help us to improve its quality. We are working on a second version. In the mean time we would like to provide you some feedback about the different issues you arose and how we are going to take your comments into account in the second version of our manuscript. Also we would like to inform you that we are going to use an updated version of the BED instance based on version 92 of Ensembl (released in April). Thus numbers provided in the article will slightly change in the next version.

- **Motivation/Rationale. The authors have correctly identified an important problem with the integration of biological data that has been addressed before, not least by the resources/tools mentioned in the manuscript (Biomart, my gene, g:Profiler amongst others). They have chosen a particularly good approach (labelled property graphs) to build the data architecture to address such a problem and one that has recently been used to great effect by the EMBL-EBI Reactome team to model data related to biological pathways. Currently this manuscript somewhat undersells the potential for the tools that have been developed. Whilst allusion is made at various points to the fact that the software developed could be used by others to develop custom resources very little is presented as to the suitability of their approach for such.**

We will address this point in the conclusion of the next version of the article by mentioning in which context BED can be used.

- **The "Abstract" states that existing resources "cannot be customised and optimised for any specific use" which is not correct and should be removed or re-worded to clarify the author's meaning.**

This statement has also been questioned by the other referee although slightly differently. We wanted to highlight the point that the way the mapping is done by most of these resources (excepted BridgeDB) cannot be customized, optimized or extended  by the user according to his knowledge or to internal, non-public or non-standard information. These tools are dedicated to a particular domain: they are focused on species, type of identifiers, and update frequencies (as stated in BridgeDB publication by van Iersel et al. (2010)). It’s convenient because ready to use but not flexible as BridgeDB or BED which allow an empowered user to focus on required information. We are going to modify this statement to make it less ambiguous in the next version.

- **Whilst the implementation presented here is focused primarily on gene level mappings it should be made clear throughout the manuscript that the general approach used could be (and indeed has been, see citation) used in other really quite different biological data modelling scenarios.**

We will mention in the introduction that graph databases, specially Neo4j, have been used to model different kind of biological data.

- **Introduction. The issue of "transitivity" is raised here, this is a complex issue for many biological data types that are far removed from the rigid structures of ontologies that commonly enforce it by definition. The meaning of "transitivity" in the context used here is not clear and warrants further explanation. This is particularly important later in the article where decisions are being made about inferring mappings where they don't exist in the data. Some such inferences are entirely logical (e.g. using HGNC ids two link gene_ids between two resources that don't map directly to each other) but others are far more complex (e.g. mapping between species). The inclusion of deprecated identifiers is excellent and will help to close a notable gap in many existing resources for which mapping older data into more recent datasets can be extremely time consuming and frustrating. The authors comment on "mapping between different scopes" is unclear and should be clarified.**

The transitivity mechanism is managed by the 2 following relationships: “corresponds_to” and “is_associated_to”. On one hand the “corresponds_to” relationships make the mapping transitive since 2 BEIDs which are connected through this kind of relationship are considered to identify the same BE through an “identifies” relationship. On the other hand a BEID which “is_associated_to” to another one does not automatically “identify” the same BE making this kind of relationship not available for indirect mappings. When the BED database is fed, the user chooses which relationship should be of type “corresponds_to” or of type “is_associated_to”. For example, in the instance we provide, cross references provided by Ensembl from Ensembl gene ID to Entrez, HGNC and Vega gene ID are considered as “corresponds_to” relationships whereas cross references to miRbase, Unigene and OMIM are considered as “is_associated_to” relationship. In Ensembl the Hs.745351 Unigene ID is mapped to ENSG00000184033 and to ENSG00000268651 Ensembl gene IDs which correspond to 2 different genes in Ensembl but also in Entrez and in HGNC and these genes are located on the same chromosome but at different positions. This Unigene identifier will be mapped to both Ensembl gene IDs but another external identifier mapped to only one of these 2 Ensembl gene ID won’t be mapped to the other (the association to Hs.745351 won’t be used indirectly).

 An identifier scope is defined by the type of BE or probe, the source of the identifiers (database or platform) and the organism. Two scopes are different when at least one of these three elements is different. Mapping is the process to identify equivalent identifiers in two different scopes. This definition comes too late and is spread in the current version of the article. We will improve it in the next version.

- **Methods. The sections "Feeding the Database" and "Querying the Database" are very brief and would benefit from much more detail about the functionality of the database creation and query system. Whilst these are covered in detail in the various pieces of documentation (including some very nice working examples) there is not enough in the manuscript itself to allow the reader to assess the available functionality.**

We will list the available functions (at least the most relevant ones) in the next version of the article.

- **Use Cases.**
- **There appears to be a discrepancy in gene counts from the Ensembl examples used in this section; the first example calls human Ensembl genes and returns 59,515 genes the second states the total number of human Ensembl genes to be 68,460.**

59,515 corresponds to the number of BE (Gene in this case). 68,460 corresponds to the number of BEIDs (Ensemble gene IDs in this case). As explained multiple BEID can identify the same BE.  In other words 59,515 BE are identified by 68,460 BEID.

- **Figure 3. illustrates a relationship graph including deprecated BEIDs. Whilst is_replaced_by is clear, it is not clear (or defined anywhere) what the meaning of is_associated_to is and how that differs from corresponds_to.  This should be clarified in the text.**

See our answer about transitivity. We will make it clearer in the next version of the article.

- **The sentence "The function guessIdOrigin..." appears out of place, unconnected to the surrounding text.**

We agree and it will be moved to another place in the next version of the article (probably in the “Additional features” section).

- **The statement "Five identifiers were only..." and the following sentence should be combined and re-worded so that the explanation as to why 5 BEIDs were uniquely found by gProfiler is clearer.**

We will refine this part to address your comment and the similar one raised by the other reviewer.

- **No validation or commentary has been presented to test the efficacy of inferences made by the query system. I would like to have seen an attempt made to check the veracity of mappings made in this way especially when the majority (c.80%) of extra Ensembl->EntrezID mappings recovered via BED were inferred**.

This kind of validation is quite difficult. We propose to use gene coordinates provided by the NCBI and Ensembl in order to compare the position on chromosomes of genes which are mapped by the different tool. Two mapped gene identifiers should have identical or similar locations. We will add these results in the next version of the article.

- **A "rough approximation" of timings for queries within BED and across other systems is not particularly informative. It would have been straightforward to automate a sampling approach to generate a mean response time (and a variance) to a defined set of query sizes/complexities to give the user a better understanding of how variable these response times are between the systems in practice. In addition, it would have been nice to see some analysis/discussion about the "scalability" of the system as this is likely to be of particular interest to end-users considering a similar modelling approach in other domains.**

We will make the analysis of mean response time with different kinds of queries and we will incorporate the results in the next version of the article. Scalability is not discussed because it highly depends on the graph database system. Here we use Neo4j for which scalability depends on the edition, community or enterprise ( https://neo4j.com/subscriptions/).

- **Figure5. The meaning of directionality here is not clear. Whilst I can see the benefit for provenance reasons i.e. a mapping from EntrezGene to RefSeq its meaning here is somewhat moot.**

This comment has also been made by the other reviewer. We will remove the arrows from the edges in figure 5 to avoid the confusion about the use of directionality to find a path between two identifiers.

# Peer review: Referee response for version 1

**DOI:** 10.5256/f1000research.15138.r31026

**Published:** 2018-03-05

**Authors:** Martina M. Summer-Kutmon, Denise Slenter

**Affiliations:** 1 Department of Bioinformatics - BiGCaT, Maastricht University, Maastricht, Netherlands

The article introduces BED a new identifier mapping tool. Using a graph database like Neo4j provides a **fast **way to query relationships between the biological entities and retrieve mappings of interest. The available source code is nicely documented and for bioinformaticians, setting up the database and running queries should be straight-forward.

 Nevertheless, there are several major issues that we would like to comment on:

- Already in the abstract, it is indicated that current tools cannot be easily customized and optimized for any specific use. It is unclear what the authors actually mean with this statement and how this is solved through BED. Further on it is also stated that current tools are generally dedicated to a particular domain, which is also true for BED. BED only focuses on gene related identifies (genes, transcripts, proteins) similar to mygene, Ensembl BioMart and g:Profiler.
- In the introduction, three main challenges are mentioned which are addressed by BED.
  - (1) Integration of mappings from different resources - very relevant but the difficult question is if transitive mappings are always biological meaningful. They can also lead to conflicting statements when resources show inconsistent relationships (we have experienced this when comparing Ensembl → UniProt and UniProt → Ensembl mappings) - how are you dealing with that? We want to state that mygene is also integrating mappings from multiple resources.
  - (2) Mapping of deprecated identifiers - this is indeed an interesting problem when analysing older datasets and the visualization in Figure 3 can be very useful when running into such issues. While you mention that BED contains all deprecated identifiers, it is not discussed why g:Profiler has five deprecated identifiers that are not in BED (Figure 4).
  - (3) Mapping scope - It is not clear why the automation of mapping between different scopes needs to be done differently and how BED is solving this. Importantly, BioMarts and mygene also provide easy ways to map between the different scopes (gene - gene / gene - protein / gene - homolog).
- Figure 3 - we believe that it would make sense to use two different edge styles for is_replaced_by and is_associated_to since they have very different meaning. Also check the layout (in this example, it looks like the blue node is placed over the edge from the purple to the light-purple node).
- Figure 5 - what do the bold borders of nodes mean in the network? Preferred identifiers? How are those selected? Additionally, when talking about the shortest relevant path, the arrows on the edges might be misleading and confusing (since there is no path from ILMN_1220595 to Q16552 taking the directionality into account). 
- The authors shortly mention the neo2R package to build the database. The functionality is not discussed in detail and it is unclear why the existing R package provided by Neo4j ( https://neo4j.com/developer/r/ was not used. Neo4j can also be easily queried from other programming languages. Are you planning to provide APIs in other languages that would allow the integration in tools other than R?
- While the conversion rate from Ensembl to Entrez Gene is very interesting, we are missing a comparison between the tools for real research examples, e.g. selection of several datasets and mapping from probe to Ensembl identifier / Entrez Gene identifier (one of the most common use cases in R workflows). This is also mentioned under the criteria for a software tool article in F1000: “The article should provide examples of suitable input data sets and include an example of the output that can be expected from the tool and how this output should be interpreted.”
- Is it possible to only include edges from certain resources when performing the identifier conversion? Or do the users need to build their own database with only those selected resources?

 

 As a final comment, we think that structure of the article is sometimes hard to follow and paragraphs are often not linked to each other. In the section “Converting identifiers” you state the following: “The aim of BED is to improve the **efficiency of identifier conversion** in a well defined context (organism, information resources of interest. . .) and not to replace biomaRt, mygene, gProfileR or other tools which provide many more features for many organisms and which should not be narrowed to this task for a complete comparison.” We believe that this efficiency, especially in the context of run time, is the key advantage of this tool and this should be made more clear in the article (abstract/intro/conclusion).

We have read this submission. We believe that we have an appropriate level of expertise to confirm that it is of an acceptable scientific standard, however we have significant reservations, as outlined above.

# Response

**Published:** 2018-04-26

**Authors:** Patrice Godard

**Affiliations:** UCB, Belgium

Thanks for having taken the time to review this article and for your constructive comments that will help us to improve its quality. We are working on a second version. In the mean time we would like to provide you some feedback about the different issues you arose and how we are going to take your comments into account in the second version of our manuscript. Also we would like to inform you that we are going to use an updated version of the BED instance based on version 92 of Ensembl (released in April). Thus numbers provided in the article will slightly change in the next version.

- **Already in the abstract, it is indicated that current tools cannot be easily customized and optimized for any specific use. It is unclear what the authors actually mean with this statement and how this is solved through BED. Further on it is also stated that current tools are generally dedicated to a particular domain, which is also true for BED. BED only focuses on gene related identifies (genes, transcripts, proteins) similar to mygene, Ensembl BioMart and g:Profiler.**

This statement has also been questioned by the other referee although slightly differently. We wanted to highlight the point that the way the mapping is done by most of these resources (excepted BridgeDB) cannot be customized, optimized or extended  by the user according to his knowledge or to internal, non-public or non-standard information. These tools are dedicated to a particular domain: they are focused on species, type of identifiers, and update frequencies (as stated in BridgeDB publication by van Iersel et al. (2010)). It’s convenient because ready to use but not flexible as BridgeDB or BED which allow an empowered user to focus on required information. We are going to modify this statement to make it less ambiguous in the next version.

- **In the introduction, three main challenges are mentioned which are addressed by BED.**
- **(1) Integration of mappings from different resources - very relevant but the difficult question is if transitive mappings are always biological meaningful. They can also lead to conflicting statements when resources show inconsistent relationships (we have experienced this when comparing Ensembl → UniProt and UniProt → Ensembl mappings) - how are you dealing with that? We want to state that mygene is also integrating mappings from multiple resources.**

The transitivity mechanism is managed by the 2 following relationships: “corresponds_to” and “is_associated_to”. On one hand the “corresponds_to” relationships make the mapping transitive since 2 BEIDs connected through this kind of relationship are considered to identify the same BE. On the other hand a BEID which “is_associated_to” to another one does not automatically “identify” the same BE making this kind of relationship not available for indirect mappings. When the BED database is fed, the user chooses which relationship should be of type “corresponds_to” or of type “is_associated_to” for each resource taken into account. For example, in the instance we provide, cross references provided by Ensembl from Ensembl gene ID to Entrez, HGNC and Vega gene ID are considered as “corresponds_to” relationships whereas cross references to miRbase, Unigene and OMIM are considered as “is_associated_to” relationship. In Ensembl the Hs.745351 Unigene ID is mapped to ENSG00000184033 and to ENSG00000268651 Ensembl gene IDs which correspond to 2 different genes in Ensembl but also in Entrez and in HGNC and these genes are located on the same chromosome but at different positions. This Unigene identifier will be mapped to both Ensembl gene IDs but another external identifier mapped to only one of these 2 Ensembl gene ID won’t be mapped to the other (the association to Hs.745351 won’t be used indirectly).

 The cross references provided by Ensembl and Uniprot between Ensembl peptide IDs and Uniprot IDs are considered as “corresponds_to” relationship in the BED instance we provide.

 If mygene integrates mapping from multiple resources it does not apply transitive mapping between Ensembl, Entrez and HGNC gene IDs (as shown in figure 4) and it does not allow the user to do it.

- **(2) Mapping of deprecated identifiers - this is indeed an interesting problem when analysing older datasets and the visualization in Figure 3 can be very useful when running into such issues. While you mention that BED contains all deprecated identifiers, it is not discussed why g:Profiler has five deprecated identifiers that are not in BED (Figure 4).**

These deprecated identifiers are not associated to any up-to-date identifier in Ensembl and as such they are not considered anymore for mapping in BED. We will develop this point in the next version of the article in order to make it clearer.

- **(3) Mapping scope - It is not clear why the automation of mapping between different scopes needs to be done differently and how BED is solving this. Importantly, BioMarts and mygene also provide easy ways to map between the different scopes (gene - gene / gene - protein / gene - homolog).**

BED use the biological relationship  between genes, transcript and peptides to convert identifiers. For example, when converting peptides identifiers from the same species it will use only mapping done at the peptide level and won’t use mapping to transcript and gene mapping. This strategy seems to be applied by biomaRt but not by mygene nor by gProfileR which map for example one Uniprot ID to all the Ensembl peptide ID coded by the same gene. For example the A6NI28 Uniprot identifier is unambiguously mapped to the ENSP00000298815 Ensembl peptide identifier by BED and biomaRt but is mapped to three additional Ensembl peptide identifiers (ENSP00000431776, ENSP00000434304 and ENSP00000435961 which are encoded by the same gene: ENSG00000165895) by mygene and gProfileR. Mapping biological entities identifier which are not genes from two different organisms using ortholog information requires at least two steps in biomaRt, mygene and gProfileR: one for find the ortholog gene and the other to find the relevant  biological entity identifier. These two steps are integrated and transparent in BED. We will add clarifying sentences in the next version of the article to address this.

- **Figure 3 - we believe that it would make sense to use two different edge styles for is_replaced_by and is_associated_to since they have very different meaning. Also check the layout (in this example, it looks like the blue node is placed over the edge from the purple to the light-purple node).**

The visNetwork library only provides 2 types of edges: solid or dash. And we would prefer not using too many colors for different types of relationships. The “is_replaced_by” and “is_associated_to” relationship can easily be differentiated using the colors of the nodes: if the nodes have the same color it is an “is_replaced_by” relationship”; if the nodes have different colors it is an “is_associated_to” relationship. In this kind of graph “is_known_as”, “identifies” (optional) or “targets” (optional) relationships can also be differentiated according to the shapes of the nodes. We will clarify this point in the figure legend.

 We will also fix the layout issue of figure 3 in the next version of the article.

- **Figure 5 - what do the bold borders of nodes mean in the network? Preferred identifiers? How are those selected? Additionally, when talking about the shortest relevant path, the arrows on the edges might be misleading and confusing (since there is no path from ILMN_1220595 to Q16552 taking the directionality into account). **

Bold borders in this figure indeed meant preferred identifiers. In the first version of the BED instance we provided (bed-ucb-human:2018.01.03), the preferred status of RefSeq transcripts and peptides is determined according to the status field provided in the gene2refseq file provided by the NCBI. The ID is preferred if the status is “MODEL”. The way to define the preferred status of Entrez gene, RefSeq transcripts and peptides will change in the next version of this instance where we will consider the assembly information also provided in the gene2refseq file: identifiers associated to non-alternative assembly will be “preferred”.

 We will remove the arrows from the edges in figure 5 to avoid the confusion about the use of directionality to find a path between two identifiers.

- **The authors shortly mention the neo2R package to build the database. The functionality is not discussed in detail and it is unclear why the existing R package provided by Neo4j (** **https://neo4j.com/developer/r/** **)** **was not used. Neo4j can also be easily queried from other programming languages. Are you planning to provide APIs in other languages that would allow the integration in tools other than R?**

Two reasons motivated our choice to develop neo2R : (i) The development of Rneo4j package was on hold for a long time period (according to github commits) and (ii) it used legacy cypher HTTP endpoint. We wanted to use the transactional HTTP endpoint as recommended in the neo4j documentation ( https://neo4j.com/docs/rest-docs/current/#rest-api-cypher). As the scope of the article is on the biological entity mapping and not on Neo4j as such, we don’t want to put emphasis on this point because we don’t think we provide strong additional value at this level.

 We do not plan to provide API in other languages but we would be happy if it is done by other developers and we would be ready to help in this frame. Indeed one of the reason to make the BED package publicly available under a GPL-3 license is to allow the community to build on it and to improve it.

- **While the conversion rate from Ensembl to Entrez Gene is very interesting, we are missing a comparison between the tools for real research examples, e.g. selection of several datasets and mapping from probe to Ensembl identifier / Entrez Gene identifier (one of the most common use cases in R workflows). This is also mentioned under the criteria for a software tool article in F1000: “The article should provide examples of suitable input data sets and include an example of the output that can be expected from the tool and how this output should be interpreted.”**

We will provide such an example in the next version. The example will be focused on the comparison of results from different experiments with different designs: different microarray platforms and organisms.

- **Is it possible to only include edges from certain resources when performing the identifier conversion? Or do the users need to build their own database with only those selected resources?**

As mentioned here-above, the conversion strategy is defined when feeding the BED database and the use of the relationships: “corresponds_to” and “is_associated_to”. At the end-user level, refinements of mapping can be achieved by using the “restricted” (which focus the mapping to non-deprecated identifiers) and the “preFilter” (which focus the mapping to preferred identifiers) parameters. Also the “getDirectProduct” and “getDirectOrigin” functions allow the user to find direct products or direct origins of molecular biology processes. For example the direct products of an Ensembl gene ID will be Ensembl transcript IDs. This is particularly useful when the user wants to focus on canonical transcription or translation events when this information is available (this is the case for Ensembl transcripts and peptides).

- **As a final comment, we think that structure of the article is sometimes hard to follow and paragraphs are often not linked to each other. In the section “Converting identifiers” you state the following: “The aim of BED is to improve the efficiency of identifier conversion in a well defined context (organism, information resources of interest. . .) and not to replace biomaRt, mygene, gProfileR or other tools which provide many more features for many organisms and which should not be narrowed to this task for a complete comparison.” We believe that this efficiency, especially in the context of run time, is the key advantage of this tool and this should be made more clear in the article (abstract/intro/conclusion).**

We adopted the structure recommended by F1000Research for a “software tool article”. Nevertheless we take note of this comment and we will try to improve the flow of the text in the next version of the article.

 We will put higher emphasis on the efficiency statement in the next version of the article.
